# Serratia marcescens Prosthetic Valve Endocarditis: Portending a Dismal Course

**DOI:** 10.7759/cureus.72936

**Published:** 2024-11-03

**Authors:** Selma Siagh, Najlaa Belharty, Hasna Rami, Nawal Doghmi, Mohamed Cherti

**Affiliations:** 1 Department of Cardiology B, Ibn Sina Hospital, Rabat, MAR

**Keywords:** infective endocarditis, non-hacek gram-negative, nosocomial and opportunistic infections, prosthetic valve infective endocarditis, serratia marcescens endocarditis, valve vegetation

## Abstract

*Serratia marcescens,* an opportunist pathogen mainly isolated in healthcare-associated infections, is a rare cause of infective endocarditis (IE) that generates an increased mortality rate compared to the usual agents. We report a case of a 70-year-old male patient who underwent a mitral valve replacement and was readmitted two months later with a high-grade continuous fever and deterioration of the general status. The diagnosis of early IE due to *S. marcescens *was established upon further investigation. The patient was treated with ertapenem and underwent surgery. Nevertheless, the prognosis was not favorable. Due to the rarity of similar presentations and the grim prognosis that* S. marcescens* IE portends, further investigation on this subject is warranted. This can aid in preventing future occurrences and help issue guidelines for therapeutic management, especially in patients with prosthetic valves.

## Introduction

Endocarditis is an inflammatory process that affects the endocardium and can have an infective or noninfective origin. It was first described by William Osler in 1885. Infective endocarditis (IE) is uncommon, with an annual incidence of 30-100 cases per million of the population. It is a deadly disease. The current in-hospital mortality rate for patients with IE is 15%-20%, with one-year mortality approaching 40% [1]. The diagnosis of IE currently relies on the Duke-International Society for Cardiovascular Infectious Diseases Criteria [2] published in 2023 and added to the 2023 European Society of Cardiology (ESC) guidelines for the management of endocarditis [3].

IE has a large number of causative organisms. Streptococci, Staphylococci, and Enterococci account for 80%-90% of all cases. IE caused by gram-negative bacteria (GNB) is usually due to Haemophilus species, *Aggregatibacter actinomycetemcomitans*, *Cardiobacterium hominis*, *Eikenella corrodens*, and Kingella species, known as HACEK organisms. It represents between 0.8% and 6.1% of all cases of IE [4]. Regarding non-HACEK gram-negative bacilli IE, it is rare, with a 2% incidence in the International Collaboration on Endocarditis (ICE) prospective cohort [1]. Furthermore, *Serratia marcescens* endocarditis is extremely rare within this category, accounting for 8%-20% of non-HACEK GNB IE [5] and 0.14% of all IE cases in the ICE study [1]. Herein, we present a complicated case of healthcare-associated endocarditis caused by *S. marcescens*.

## Case presentation

A 70-year-old male patient consulted for a prolonged fever and an alteration of the general status. The patient had a history of very severe mitral stenosis and had undergone two months earlier a mitral valve replacement by a bileaflet mechanical valve, with no postoperative complications. He had no other medical history, notably no previous history of drug abuse or immunodeficiency.

**Figure 1:**
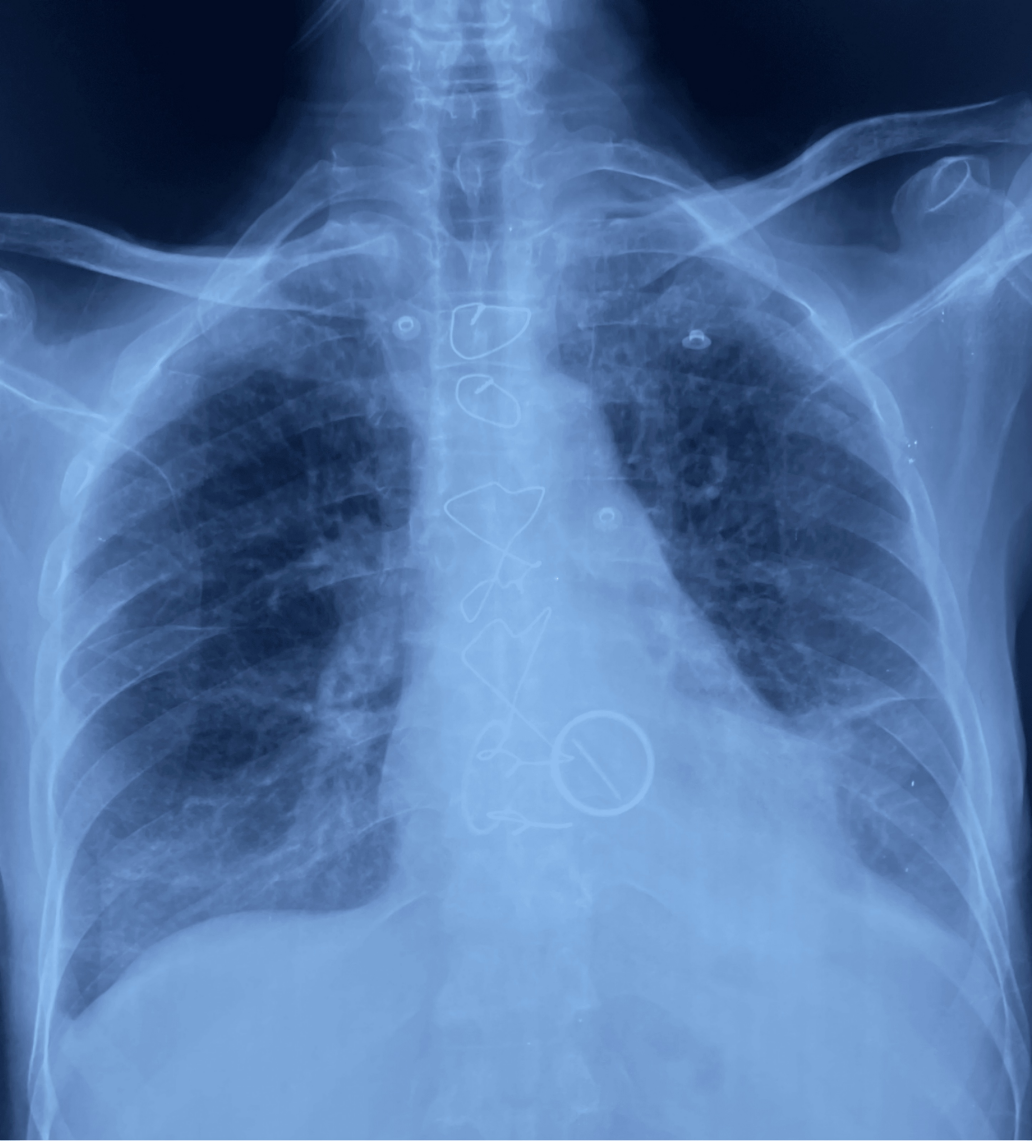
Chest X-ray showing no evident opacity

After his discharge from the cardiovascular surgery department, the patient reported the occurrence of an intermittent fever, getting progressively worse, eventually leading to his admission to the cardiology department. Upon first medical contact, the patient exhibited confusion, demonstrated by a Glasgow Coma Score of 14, and presented with a fever of 39.2°C. He was hemodynamically stable with a blood pressure of 115/66 mmHg and a heart rate of 100 bpm. He had a respiratory rate of 16 cpm and a saturation of 97% on room air. Cardiac auscultation found prosthetic mitral valve sounds and a systolic murmur of mitral regurgitation. The electrocardiogram showed atrial fibrillation, and the chest X-ray revealed no evident opacity (Figure 1).

Initial laboratory testing (Table 1) revealed hemolytic anemia with hemoglobin at 9.1 g/dL (normal range, NR: 13-16.5), neutrophilic leukocytosis with white blood cells at 32,900 elements/mm^3^ (NR: 4,000-10,000), hepatic cytolysis with aspartate aminotransferase at 162 international units (IU)/L (NR: 5-34), and alanine transaminase at 66 IU/L (NR: 0-55), as well as cholestasis with alkaline phosphatase at 261 IU/L (40-150 IU/L), GGT at 203 IU/L (NR: 9-36), and hyperbilirubinemia at 60 mg/L (NR: 2-12) mainly conjugated with 40 mg/L (NR: 0-5), no electrolyte imbalance, and a normal renal function. Finally, the patient had elevated C-reactive protein at 281 mg/L (NR < 6 mg/L) and procalcitonin at 4 ug/L (NR < 0.05).

**Table 1:** Initial laboratory findings MCV: mean corpuscular volume; MCHC: mean corpuscular hemoglobin concentration; IU: international unit

Laboratory test	Result	Reference value
Hemoglobin	9.1 g/dL	13-16.5
MCV	85 μm^3^	80-100
MCHC	34%	32-36
Reticulocytes	251,000/mm^3^	<120,000
White blood cells	32,900/mm^3^	4,000-10,000
Neutrophils	28,400/mm^3^	1,500-7,000
Platelets	171,000/mm^3^	150,000-400,000
Sodium	139 mmol/L	135-145
Potassium	4.6 mmol/L	3.5-5
Chlorine	98 mmol/L	98-107
Bicarbonates	18 mmol/L	22-26
Urea	0.46 g/L	0.15-0.55
Creatinine	10 mg/L	7.2-12.5
Aspartate transaminase	162 IU/L	5-34
Alanine transaminase	66 IU/L	0-55
Alkaline phosphatase	261 IU/L	40-150
Gamma-glutamyl transferase	203 IU/L	9-36
Bilirubin	60 mg/L	2-12
Conjugated bilirubin	40 mg/L	0-5
C-reactive protein	281 mg/L	<6
Procalcitonin	4 μg/L	<0.05

**Figure 2:**
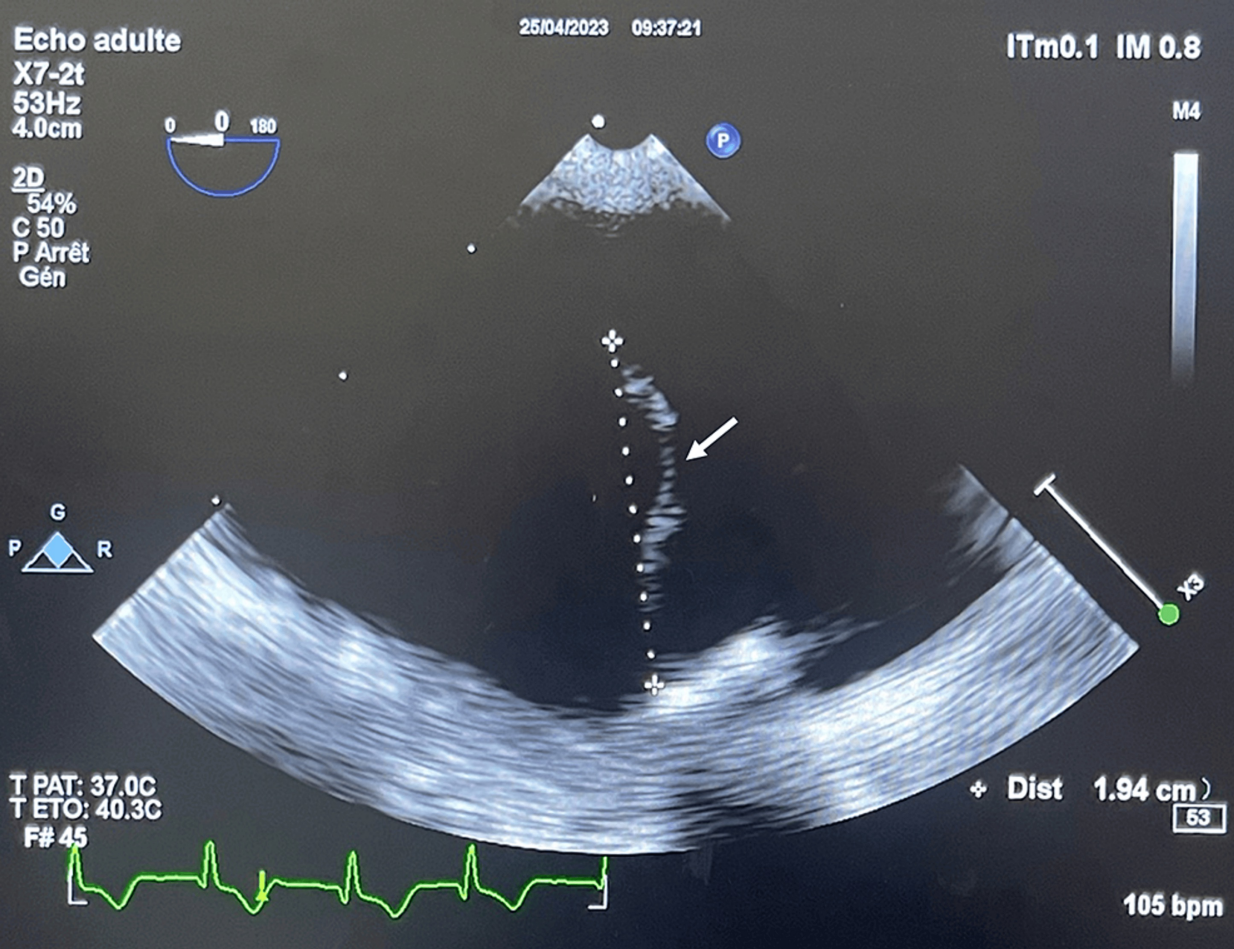
Mid-esophageal view on transesophageal echocardiogram showing serpentine vegetation affixed to the prosthetic valve

An emergency transthoracic echocardiogram (TTE) showed a new moderate paraprosthetic mitral regurgitation and mobile elements on the atrial side of the prosthetic valve. Therefore, a transesophageal echocardiogram (TEE) was performed and showed several mobile elements on the atrial side of the mitral valve, the largest of which was 20 mm long (Figure 2), as well as two small elements on the ventricular side of the right anterior aortic cusp. It also showed a prosthetic mitral valve dehiscence with moderate paraprosthetic leak (Videos 1, 2).

**Video 1:** Transesophageal echocardiogram showing dehiscence of the mitral prosthetic valve, exhibiting the distinctive rocking motion

**Video 2:** Transesophageal echocardiogram showing on color Doppler an eccentric paravalvular regurgitation

According to these findings, IE was highly probable, and the patient was empirically started on intravenous vancomycin (1 g every eight hours) and gentamicin (180 mg per day). A computed tomography (CT) scan of the brain and the thoraco-abdomino-pelvic region was performed, which revealed a splenic infarction. Four sets of aerobic and anaerobic blood cultures (Table 2) and a urine culture (Table 3) were positive for *S. marcescens, *and* *antimicrobial susceptibility testing was performed (Table 4).

**Table 2:** Aerobic and anaerobic blood cultures

Variable	Result
Aerobic blood culture	Positive
Time to positivity	7 hours and 20 minutes
Organism	S. marcescens
Anaerobic blood culture	Positive
Time to positivity	6 hours and 53 minutes
Organism	S. marcescens

**Table 3:** Urine culture NR: normal range; NA: not applicable

Variable	Result	Reference value
White blood cells	22 elements/mm^3^	NR < 10
Red blood cells	22 elements/mm^3^	NR < 15
Epithelial cells	0 elements/mm^3^	NR < 10
Crystals	Absence	NA
Yeast	Absence	NA
Culture	S. marcescens	NA

**Table 4:** Antibiogram profile of the S. marcescens isolates

Antibiotic tested	Result
Aminoglycosides	
Amikacin	Resistant
Gentamicin	Resistant
Carbapenems	
Ertapenem	Susceptible
Imipenem	Susceptible
Cephalosporins	
Cefalotin	Resistant
Cefoxitin	Resistant
Ceftriaxone	Susceptible
Ceftazidime	Susceptible
Penicillins	
Ampicillin	Resistant
Ticarcillin	Susceptible
Amoxicillin-clavulanate	Resistant
Ticarcillin-clavulanate	Susceptible
Piperacillin-tazobactam	Susceptible
Nitrofurans	
Nitrofurantoin	Resistant
Quinolons	
Ciprofloxacin	Susceptible
Nalidixic acid	Resistant

The therapeutic regimen was then adapted to the antibiogram profile: we discontinued the prior empiric antibiotics and initiated ertapenem (2 g per day). Follow-up blood cultures performed 48 hours later remained positive, and on the third day of hospitalization, the patient was transferred to the surgical ward, where a complete debridement and valve replacement were performed. Nevertheless, the outcome was unfavorable, with uncontrolled sepsis, hemodynamic instability, and multiple organ dysfunction, ultimately leading to the patient's death.

## Discussion

*S. marcescens* is a motile gram-negative bacillus that is a facultative anaerobe belonging to the family Yersiniaceae in the order Enterobacterales. It is a ubiquitous bacteria that can be isolated in water, soil, sewage, and food [6]. Until recent decades, *S. marcescens* was considered a saprophytic germ. The first report associating it with IE and other diseases was published by Wheat et al. in 1951 [7]. It is now recognized as a human pathogen responsible for a wide range of infections, including pneumonia, urinary tract infections, and endocarditis. These infections mainly affect patients with a compromised immune system, as *S. marcescens* is typically an opportunistic pathogen. Moreover, they occur in the nosocomial setting, where *S. marcescens* accounts for 1%-2% of all nosocomial infections and is primarily isolated in respiratory tract infections, urinary tract infections, and surgical site infections [6].

Regarding specifically IE due to *S. marcescens*, it has been historically associated with intravenous drug use. This idea is traceable to two reports, published in 1976 and 1980 in San Francisco, describing two clusters of cases of IE due to *S. marcescens* [8,9]. Currently, however, healthcare exposure is considered a common source of infection. In fact, in the ICE prospective cohort study, 57% of the cases of non-HACEK gram-negative bacilli endocarditis were healthcare-associated [1].

The main identified risk factors for IE due to Serratia spp. include invasive procedures, implanted endovascular devices (prosthetic valves, permanent pacemakers, or defibrillators), and comorbid conditions [1,5,10]. Moreover, the infection is most frequently acquired through the genitourinary tract [5,10], while the nonoral gastrointestinal tract is also reported as an infectious entry point [5].

In relation to our patient, we concluded that it was a case of healthcare-associated endocarditis. Furthermore, given the positive urine culture, the entry point may have been urinary, especially since the patient had been catheterized during his stay in the cardiovascular surgery department. This aligns with the epidemiological data mentioned above. In addition, in our case, the IE was complicated locally by the detachment of the prosthetic valve and systemically by septic shock and splenic embolization. The largest vegetation observed on transesophageal echocardiography measured 20 mm, which aligns with data from an Italian prospective multicenter cohort suggesting that vegetations are typically larger in patients with IE caused by gram-negative bacilli [10].

Furthermore, prosthetic valve endocarditis (PVE) due to *S. marcescens* is very rare. Only three cases were reported in the English literature: one case of early PVE reported by Lyall et al. [11] and two cases of late PVE reported by De Silva et al. [12] and Kochi et al. [13] (Table 5).

**Table 5:** Published cases (English language) of prosthetic valve infective endocarditis due to S. marcescens since 1997 PVE: prosthetic valve endocarditis; NS: not specified

Study	Age/sex	Heart prosthetic	Time between surgery and PVE	Location	Extracardiac complications	Sample in which S. marcescens was isolated	Treatment	Outcome
Lyall et al. [11]	65 years old, female	Bentall procedure + coronary artery bypass graft surgery	14 days	Aortic valve	Bilateral endophthalmitis	Blood cultures, and aqueous and vitreous samples	Meropenem and gentamycin	Survived blind
De Silva et al. [12]	67 years old, male	Dual chamber pacemaker	6 years	Pacing wire (right heart endocarditis)	None	Blood culture and culture from the pacemaker lead	Meropenem and gentamycin	Survived
Kochi et al. [13]	57 years old, female	Mitral valve replacement	32 months	Mitral valve	Hemolytic anemia	Blood culture	NS	NS

With regard to therapeutic care, *S. marcescens *endocarditis presents a major challenge due to its multidrug resistance. In fact, *S. marcescens* strains are resistant to a wide range of antibiotics, including narrow-spectrum penicillins (ampicillin, amoxicillin, amoxicillin-clavulanate, and ampicillin-sulbactam), first and second-generation cephalosporins, macrolides, nitrofurans, and colistin. The resistance of *S. marcescens* to antibiotics is mainly reliant on antibiotic-modifying enzymes, mainly AmpC β-lactamases. Furthermore, some strains of *S. marcescens *express extended spectrum β-lactamases that can hydrolyze broad-spectrum agents such as cefotaxime, ceftazidime, and cefepime. Even more alarmingly, a number of *S. marcescens* isolates have acquired carbapenem-inactivating enzymes [6].

Thereby, given the rarity of *S. marcescens *endocarditis and its wide resistance profile, the American Heart Association and the ESC make no specific antimicrobial recommendations for its management. More broadly, for non-HACEK gram-negative bacilli endocarditis, they recommend a long-term antibiotic therapy of at least six weeks with a combination of a β-lactam (penicillin, cephalosporin, or carbapenem) and either an aminoglycoside or a fluoroquinolone or cotrimoxazole [3,14]. Besides, antibiotic sensitivity tests should be considered, given the various mechanisms of antibiotic resistance. In all the cases of PVE due to *S. marcescens* reported in the literature, including ours, the antibiotic regimen included a carbapenem and gentamycin. Nevertheless, the emergence of carbapenemase-mediated resistance in *S. marcescens* suggests that judicious use of these agents should be advised.

The case we reported is notable for involving an unusual germ (*S. marcescens)* in a PVE. It poses management challenges for several reasons, primarily due to the multidrug-resistant profile of *S. marcescens* and the rapid progression of the disease. The initial TTE and later TEE revealed multiple vegetations, the largest measuring over 10 mm, along with moderate prosthetic valve dysfunction. Moreover, there was no clinical evidence of systemic embolisms. Therefore, in accordance with the ESC guidelines [3] and after consulting the surgical department, the decision was made to initiate antibiotic treatment and perform a whole-body and brain CT scan to search for systemic embolisms. With established systemic embolisms (splenic infarcts) and persistent positive blood cultures, urgent surgery (i.e., within three to five days) was performed on day three of hospitalization, in accordance with the guidelines [3]. Despite appropriate antibiotic therapy and surgical intervention, the evolution was not favorable.

## Conclusions

PVE induced by *S. marcescens* is a rare yet fatal condition that requires prompt diagnosis and appropriate antibiotic therapy to be managed effectively. Therapeutic options remain scarce owing to this pathogen's highly resistant profile. Key takeaways from this case include the fact that *S. marcescens* should be regarded as a predictor of poor outcomes, prompting practitioners to assess the indication for surgery at an earlier stage. Finally, this case highlights more broadly the importance of preventing healthcare-associated infections and antibiotic resistance.

## References

[1] Murdoch DR, Corey GR, Hoen B (2009). Clinical presentation, etiology, and outcome of infective endocarditis in the 21st century: the International Collaboration on Endocarditis-Prospective Cohort Study. Arch Intern Med.

[2] Fowler VG, Durack DT, Selton-Suty C (2023). The 2023 Duke-International Society for Cardiovascular Infectious Diseases criteria for infective endocarditis: updating the modified Duke criteria. Clin Infect Dis.

[3] Delgado V, Ajmone Marsan N, de Waha S (2023). 2023 ESC guidelines for the management of endocarditis. Eur Heart J.

[4] Tleyjeh IM, Steckelberg JM, Murad HS (2005). Temporal trends in infective endocarditis: a population-based study in Olmsted County, Minnesota. JAMA.

[5] Morpeth S, Murdoch D, Cabell CH (2007). Non-HACEK gram-negative Bacillus endocarditis. Ann Intern Med.

[6] Tavares-Carreon F, De Anda-Mora K, Rojas-Barrera IC, Andrade A (2023). Serratia marcescens antibiotic resistance mechanisms of an opportunistic pathogen: a literature review. PeerJ.

[7] Wheat RP, Zuckerman A, Rantz LA (1951). Infection due to chromobacteria; report of eleven cases. AMA Arch Intern Med.

[8] Mills J, Drew D (1976). Serratia marcescens endocarditis: a regional illness associated with intravenous drug abuse. Ann Intern Med.

[9] Cooper R, Mills J (1980). Serratia endocarditis. A follow-up report. Arch Intern Med.

[10] Falcone M, Tiseo G, Durante-Mangoni E (2018). Risk factors and outcomes of endocarditis due to non-HACEK Gram-negative bacilli: data from the prospective multicenter Italian endocarditis study cohort. Antimicrob Agents Chemother.

[11] Lyall DA, Gregory ME, McDonnell J, De Villiers F, Tejwani D (2013). Bilateral endogenous Serratia marcescens endophthalmitis secondary to endocarditis following cardiac surgery. Scott Med J.

[12] De Silva K, Fife A, Murgatroyd F, Gall N (2009). Pacemaker endocarditis: an important clinical entity. BMJ Case Rep.

[13] Kochi K, Yokote Y, Kyo S, Ueda K, Omoto R (1997). Serratia marcescens prosthetic mitral valve endocarditis associated with hemolytic anemia. [Article in Japanese]. Nihon Kyobu Geka Gakkai Zasshi.

[14] Baddour LM, Wilson WR, Bayer AS (2015). Infective endocarditis in adults: diagnosis, antimicrobial therapy, and management of complications: a scientific statement for healthcare professionals from the American Heart Association. Circulation.

